# Late diagnosis of Kartagener syndrome in a 38-year-old female presenting with palpitations in a resource-limited emergency department

**DOI:** 10.1093/omcr/omaf233

**Published:** 2025-11-26

**Authors:** Ömer Atlı

**Affiliations:** Department of Emergency Medicine, Hazro State Hospital, Diyarbakır, Turkey

**Keywords:** Kartagener syndrome, Dextrocardia, situs inversus, emergency medicine, late diagnosis

## Abstract

Kartagener syndrome, a subset of primary ciliary dyskinesia, is typically diagnosed in childhood due to its classic triad of situs inversus, chronic sinusitis, and bronchiectasis. We report a compelling case of a 38-year-old woman from a remote village who presented to our emergency department with palpitations, dyspnea, and reflux. All symptoms resolved with rest and PPIs. Routine investigations revealed dextrocardia on ECG and chest radiography. Despite a limited history of hospital visits, she had a known history of nasal polyps, chronic cough with mucus expectoration, and recurrent bronchitis. A clinical diagnosis of Kartagener syndrome was made based on clinical features and basic diagnostic tools. This case underscores the importance of maintaining diagnostic vigilance in adult patients and highlights the potential for accurate diagnosis even in resource-constrained settings. It emphasizes how physical examination and plain radiography can reveal significant diagnostic clues, even in the absence of advanced investigations.

## Introduction

Kartagener syndrome is a rare autosomal recessive disorder affecting mucociliary clearance, with an incidence of approximately 1 in 30 000. It is often diagnosed in childhood, typically due to recurrent respiratory infections, situs inversus, or infertility. Adult diagnoses are uncommon and typically arise in more developed healthcare environments with advanced diagnostics. This case presents an unusual example of Kartagener syndrome diagnosed in a 38-year-old woman in a resource-limited rural emergency department, based on basic clinical assessment and plain-film imaging.

## Case presentation

A 38-year-old woman from a remote village presented to our emergency department with palpitations, dyspnea, and symptoms of gastroesophageal reflux. Her symptoms improved with rest and administration of a proton pump inhibitor. On further questioning, she described a long-standing history of chronic cough with thick mucus expectoration, occasionally blood-streaked. She reported that she ‘feels and hears the sputum in her chest’ particularly when laughing. She had previously been diagnosed with nasal polyps and recurrent bronchitis despite infrequent hospital visits.

She also reported persistent nasal congestion despite undergoing surgery for nasal polyps the year before. Hearing difficulties, especially with loud voices, were also present. She noted episodic dyspnea on exertion and environmental triggers such as cigarette smoke, dust, and fumes. She reported chronic fatigue and sleep disruption due to nasal obstruction, frequent snoring, and mouth breathing.

Her menstrual cycle was regular but occurred every 20 days, and often painful enough to require analgesics. She was single, never married, and had never attempted pregnancy. Of note, her first name is traditionally male in Turkish culture, echoing the reversed theme present in her underlying condition.

Vital signs on presentation were within normal limits. On auscultation, the cardiac apex beat and heart sounds were clearly localized on the right hemithorax. Bilateral crackles were heard in the lower lung fields. An ECG revealed low-voltage QRS complexes in V5–V6, and inverted P and T waves in lead I, prompting further evaluation. A follow-up ECG with right-sided chest leads demonstrated more appropriate voltage in the precordial leads. An additional ECG with reversed right and left arm leads normalized the appearance of lead I. A chest radiograph revealed a right-sided cardiac silhouette, and a right-sided gastric bubble consistent with situs inversus. An erect abdominal film further supported the reversal of visceral anatomy.

She was later referred to a pulmonologist and underwent a thoracoabdominal CT, which confirmed situs inversus totalis. She was counseled about the condition and advised for respiratory and ENT follow-up. The diagnosis initially caused emotional distress to the patient, as she was shocked and scared, but she was reassured that the condition is congenital and not life-threatening in itself. She expressed relief after understanding the implications and appreciated the clarity it brought to her longstanding symptoms.

## Discussion

This case illustrates the diagnostic value of basic tools such as ECG and chest radiography in a resource-limited setting. The patient lived in a rural village with limited access to care, despite having significant long-standing symptoms. Unlike many published cases relying on high-resolution CT or electron microscopy, this case was diagnosed using fundamental bedside and radiographic techniques.

Her history fulfilled the triad of Kartagener syndrome: situs inversus (confirmed radiographically), chronic sinusitis and nasal polyps (ENT history), and chronic productive cough with occasional haemoptysis (suggesting bronchiectasis). Her description of chronic fatigue, poor sleep, and impaired hearing added further clinical depth.

The patient’s presentation with palpitations and reflux—rather than classic respiratory symptoms—highlights the variability of Kartagener syndrome in adults. The initial suspicion was raised due to abnormal ECG findings including lead I inversion and low voltages in the lateral leads. Importantly, bedside findings such as the altered location of the cardiac apex beat and heart sounds on the right side should prompt suspicion for dextrocardia and further investigation.

Diagnosing Kartagener syndrome in adulthood presents a challenge due to its variable and often non-specific presentation. Adult patients may not report classical respiratory symptoms or may attribute them to more common conditions. Prognostically, earlier diagnosis allows for interventions that can mitigate respiratory complications and improve quality of life. Late diagnosis may lead to chronic damage and reduced pulmonary function.

The learning point of this case is the importance of maintaining a high index of suspicion and utilizing thorough physical examination combined with basic investigations like chest X-ray and ECG. Even in a setting without access to CT or genetic testing, clinicians can make a confident diagnosis of a rare condition through attentive clinical observation and history taking.

## Conclusion

Kartagener syndrome can be diagnosed even in remote and resource-limited emergency settings through astute clinical observation and judicious use of basic diagnostics. This case reinforces the need to consider rare congenital disorders in adults with unexplained chronic symptoms and underscores the role of clinical acumen in the absence of advanced investigations.

### Patient perspective

‘When I laugh, I feel and hear the sputum in my chest. It’s full. I’ve always felt tired, like I’m stuck to the ground and can’t fully get up and go,’ the patient stated. Initially, she felt shocked and frightened by the diagnosis, but after understanding it is not a disease but a congenital variation, she felt reassured and relieved.

### Confidentiality note

To preserve patient confidentiality, no real names or identifiable details are used. All data has been anonymised. Written informed consent was obtained for publication.

### Patient consent

Written informed consent was obtained from the patient for the publication of this case report, including anonymised diagnostic images.

**Figure 1:**
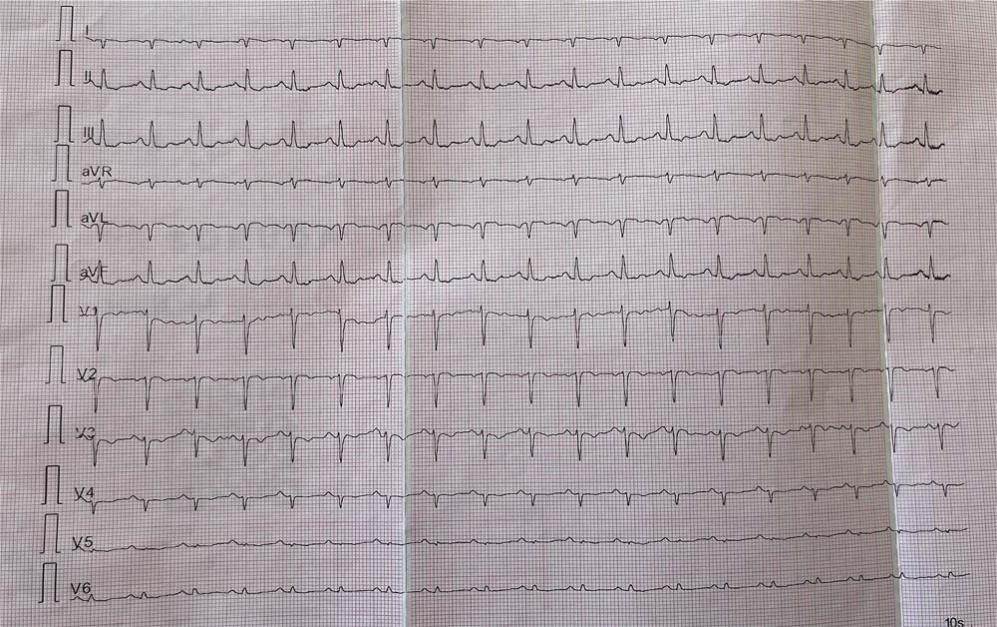
Standard 12-Lead Electrocardiogram (Anterior Chest Leads): Electrocardiogram recorded with standard lead placement demonstrating inverted P and T waves in lead I, with low voltage QRS complexes in leads V5–V6, suggestive of dextrocardia. The abnormal axis and poor R wave progression prompted further evaluation.

**Figure 2:**
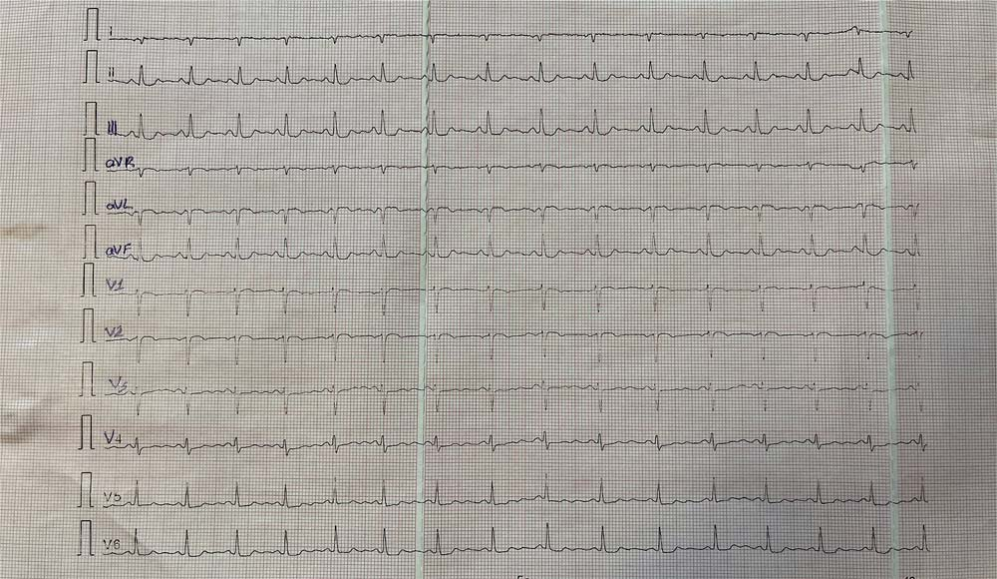
Right-Sided Electrocardiogram: ECG recorded with precordial leads placed on the right side of the chest, showing restored R-wave progression and improved QRS amplitude. These findings support a diagnosis of dextrocardia by correcting the lead misalignment seen in standard 12-lead placement.

**Figure 3:**
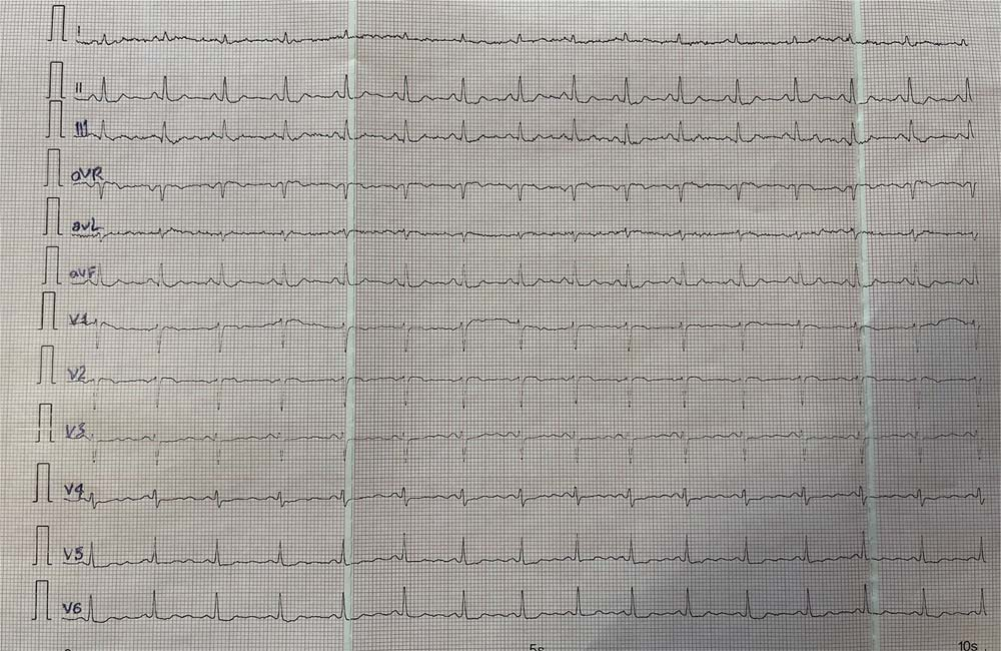
Right-Sided ECG with Arm Lead Reversal: ECG recorded with both right-sided precordial leads and reversal of right and left arm leads, resulting in normalization of lead I morphology and axis orientation. This maneuver aids confirmation of true dextrocardia, distinguishing it from technical errors such as limb lead misplacement.

Figures 1–3 illustrate standard and modified ECG findings consistent with dextrocardia.

**Figures 4:**
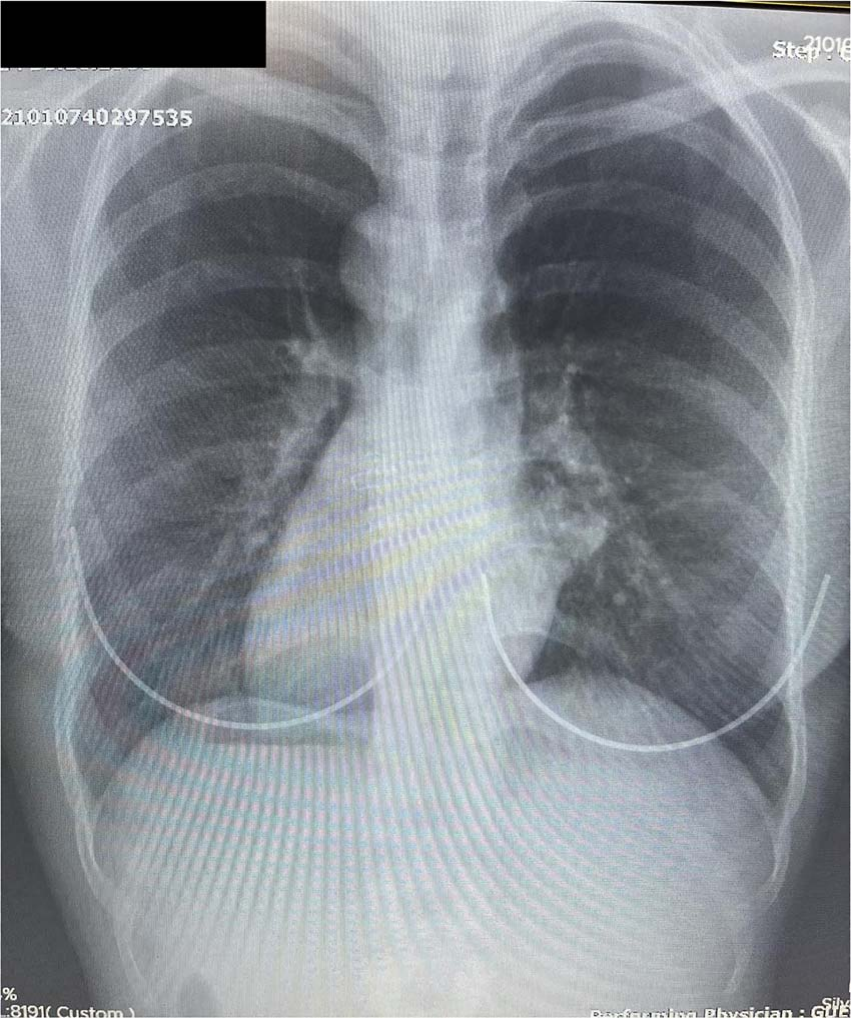
Chest Radiograph (Posteroanterior View): Chest X-ray showing dextrocardia with a right-sided cardiac apex and normal positioning of the mediastinum toward the right. The gastric bubble is visible on the right, supporting the diagnosis of situs inversus totalis.

**Figure 5:**
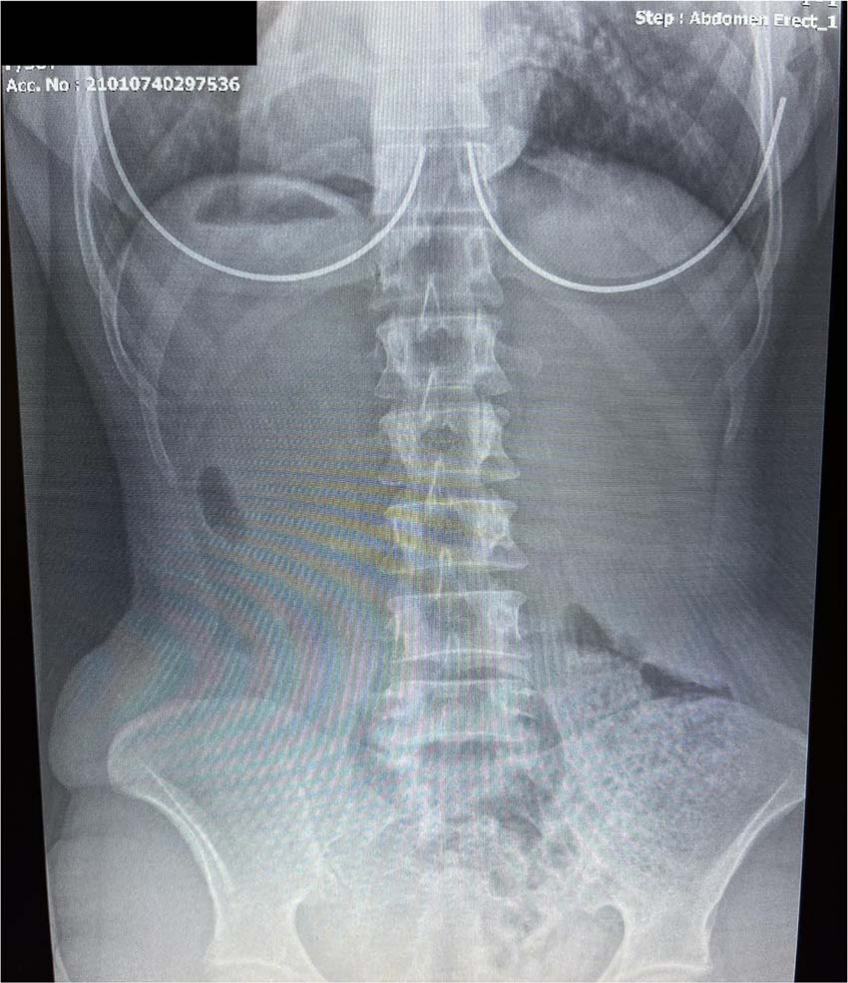
Erect Abdominal Radiograph: Abdominal X-ray (erect view) demonstrating a right-sided gastric air bubble, a hallmark feature of situs inversus totalis. This imaging finding, when correlated with dextrocardia, supports the diagnosis of Kartagener syndrome.

Figures 4 and 5 display the chest and erect abdominal X-rays demonstrating situs inversus. Together, these confirm the diagnosis using simple bedside tools, reinforcing the learning point.
